# MicroRNA-124 enhances response to radiotherapy in human epidermal growth factor receptor 2-positive breast cancer cells by targeting signal transducer and activator of transcription 3

**DOI:** 10.3325/cmj.2016.57.457

**Published:** 2016-10

**Authors:** Ying Fu, Jianping Xiong

**Affiliations:** 1 Department of General Surgery, the First Affiliated Hospital of Nanchang University, Nanchang, China; 2 Department of Oncology, the First Affiliated Hospital of Nanchang University, Nanchang, China

## Abstract

**Aim:**

To determine whether microRNA (miR)-124 enhances the response to radiotherapy in human epidermal growth factor receptor 2 (HER2)-positive breast cancer cells by targeting signal transducer and activator of transcription 3 (Stat3).

**Methods:**

miR-29b expression was measured in 80 pairs of breast tumor samples and adjacent normal tissues collected between January 2013 and July 2014. Activity changes of 50 canonical signaling pathways upon miR-124 overexpression were determined using Cignal Signal Transduction Reporter Array. Target gene of miR-124 was determined using Targetscan and validated by Western blotting and dual-luciferase assay. Cell death rate was assessed by propidium iodide (PI)/ Annexin V staining followed by flow cytometry analysis. Stat3 and miR-124 expression was further measured in 10 relapsed (non-responder) and 10 recurrence-free HER2-positive breast cancer patients.

**Results:**

MiR-124 expression was down-regulated in HER2 positive breast cancers compared with normal tissues, and was negatively associated with tumor size. MiR-124 overexpression in HER2 positive breast cancer cell line SKBR3 significantly reduced the activity of Stat3 signaling pathway compared with control transfection (*P* < 0.001). Bioinformatic prediction and function assay suggested that miR-124 directly targeted Stat3, which is a key regulator of HER2 expression. MiR-124 overexpression down-regulated Stat3 and potently enhanced cell death upon irradiation. Consistently, chemical inhibitor of Stat3 also sensitized HER2-positive breast cancer cells to irradiation. Moreover, increased Stat3 expression and reduced miR-124 expression were associated with a poor response to radiotherapy in HER2-positive breast cancers.

**Conclusions:**

Weak miR-124 expression might enhance Stat3 expression and radiotherapy resistance in HER2-positive breast cancer cells.

Breast cancer is the most common female malignancy worldwide, with nearly 1.7 million cases diagnosed each year (1). The etiology of this malignant disease is complex. Based on its characteristics, treatment regimens, and clinical outcomes, breast cancer is commonly categorized into 5 subtypes, including luminal A, luminal B, basal-like, HER2-positive, and normal-like (2). Of note, HER2-positive breast cancer, which accounts for about 25% of all cancer cases, is associated with a poor prognosis and a high recurrence rate (1,3). Accordingly, understanding the molecular mechanisms underlying the etiology of different breast cancer subtypes is critical to the development of novel therapies and improvement of clinical outcomes of breast cancer patients.

MicroRNAs (miRNAs) are a 21-25 long class of small non-protein coding RNA, which are critical in regulating gene expression by degrading the target mRNAs and/or inhibiting translation (4,5). By targeting different genes with various oncogene or tumor-suppressor functions, miRNAs are playing a wide range of roles in regulating proliferation, apoptosis, metastasis, and resistance to various therapies. Consistent with their critical roles in tumorigenesis, single-nucleotide variants and copy-number changes in miRNA genes are frequently observed in various cancers (5-8). Abnormal transcription and defects in miRNA biogenesis may also influence the production of cancer-related miRNAs (9). Moreover, as miRNAs could be selectively packaged into exosomes and differentially secreted, circulating miRNAs have therefore emerged as a sensitive marker for early cancer detection.

Accumulating evidence has supported the role of miR-124 as an important tumor suppressor, and reduced miR-124 expression or its lack was reported in several types of human cancers. Notably, it has been reported that poor expression of microRNA-124 is a prognostic biomarker of breast cancer patients,while miR-124 suppresses multiple steps of breast cancer metastasis by targeting a cohort of pro-metastatic genes (10-14) However, the role of miR-124 in HER2-positive breast cancer remains largely unknown. Therefore, the aim of this study was to investigate the association between miR-124 expression and different pathological and clinical characteristics of patients with HER2-positive breast cancer. We also identified Stat3 as the major target of miR-124. It has been shown that STAT3-survivin signaling mediated a poor response to radiotherapy in HER2-positive breast cancers (15). We thus hypothesized that weakly expressed miR-124 might enhance Stat3 expression and activity, contributing to radiotherapy resistance in these cells.

## Materials and methods

### Tumor tissues and cell culture

The study design was revised and approved by the Institutional Ethics Committee of the First Affiliated Hospital of Nanchang University and informed consent was obtained from each patient. Pathological and clinical information on these patients is shown in Table 1. Only patients who did not receive any therapy before recruitment to this research were included in the study. Between January 2013 and July 2014, 80 tumor samples and matching adjacent normal tissues from the same patients collected in the First Affiliated Hospital of Nanchang University. Samples were stored in RNAlater solution (Ambion, Chelmsford, MA, USA), confirmed by pathologists and then stored at -80°C until total RNA extraction. For radiation therapy resistance analysis, tumor samples were obtained from an independent cohort of 20 patients treated by curative surgery and adjuvant radiotherapy between February 2010 and July 2014. The human breast cancer cell lines SK-BR-3, T47D, and MCF7 obtained from the Shanghai Institute of Biological Sciences were cultured in DMEM (Thermo Fisher, Chelmsford, MA, USA) containing 10% heat-inactivated fetal bovine serum (Thermo Fisher) and penicillin/streptomycin (Thermo Fisher), at 37°C with 5% CO_2_.

**Table 1:** Expression of microRNA-124 and clinicopathological factors in 80 breast cancer patients*

Variable	No. of cases	Mean expression	*P*
Samples			
tumor	80	1.00 ±0.67	**0.0016^†^**
normal	80	2.65±0.91	
Age (years)			
<50	16	1.12±0.56	0.36
>50	64	0.95±0.51	
Tumor size (cm)			
<5	33	0.81±0.59	**0.033^†^**
>5	47	1.15±0.52	
Stage			
I	11	0.87±0.66	0.061
II-IV	69	1.02±0.59	
Histology			
infiltrating duct	51	0.98±0.65	0.58
infiltrating lobular	29	1.06±0.61	
Lymph node metastasis			
absent	25	1.09±0.71	0.45
present	55	0.95±0.57	
Estrogen-receptor status			
positive	52	0.95±0.55	0.67
negative	28	1.11±0.63	
Progesterone receptor status			
positive	39	1.03±0.55	0.58
negative	41	0.97±0.59	
Human epidermal growth factor receptor 2 status			
positive	32	0.81±0.51	**0.024^†^**
negative	48	1.13±0.68	

*Data are presented as means ± standard deviation. Mean expression of all tumor samples was normalized to 1.00.

†*P* < 0.05, t-test.

### RNA extraction and real-time polymerase chain reaction (PCR)

We used Trizol reagent (Thermo Fisher) to extract total RNA from different tumor samples and cell lines. After extraction, RNA levels were analyzed for concentrations and purity using UV/Vis spectroscopy at 230, 260, and 280 nm. Total RNA was used as the template for reverse transcription using the TaqMan Reverse Transcription Kit (Applied Biosystems, Carlsbad, CA, USA) with probes for miR-124 and U6 as the internal control. Quantitative PCR assays for each sample were run in triplicate using the TaqMan Real-Time PCR Kit (Applied Biosystems).

### Transfection of miR-124 duplex

MiR-124 duplex and the control oligo were obtained from Shanghai GenePharma Company (Shanghai, China) and were transfected using Lipofectamine 2000 (Thermo Fisher) at a final concentration of 50 nM. The sequences of the miRNA duplex were miR-124 sense: 5′- UAAGGCACGCGGUGAAUGCCA-3′; miR-124 antisense: 3′-UAAUUCCGUGCGCCACUUACG-5′. Control sense: 5′-AGUACUGCUUACGAUACGGTT-3′; Control antisense: 3′-TTUCAUGACGAAUGCUAUGCC-5′.

### MiRNA target analysis and luciferase assay

Putative genes that are miR-124 targets were analyzed using TargetScan V7.1 (*http://www.targetscan.org/vert_71/).* To generate the luciferase reporter vector, a 312 bp human Stat3 gene 3′-untranslated regions (3′-UTR) segment encompassing the predicted miR-124 binding sites was PCR-amplified and subcloned into the pGL3 luciferase plasmid. The mutant construct was made with the QuikChange site-directed mutagenesis kit (Stratagene, La Jolla, CA, USA). Dual luciferase activity assay was performed according to the instruction manual (Promega, Madison, WI, USA).

### Signal Transduction Reporter Array

Activity changes of 50 canonical signaling pathways in response to miR-124 overexpression were determined using Signal Transduction Reporter Array (Qiagen, Cambridge, MA, USA). Cells cultured in 6-well plate were transfected with miR-124 or scramble control for 36 h, and then plated on the Cignal Signal Transduction Reporter Array for transfection with a mixture of a transcription factor responsive firefly luciferase reporter and a constitutively expressing Renilla construct in each well. The relative activity of each pathway was determined by luciferase/Renilla and normalized by untreated controls.

### Western blotting

10 μg proteins of each sample were separated on SDS-PAGE and subjected to immunoblotting using HER2 (1:1000, Cell signaling), p-Stat3 (1:1000, Abcam, Cambridge, MA, USA), Stat3 (1:1000, Abcam), Estrogen receptors (1:2000, Proteintech, Rosemont, IL, USA), and Tubulin (1:5000, Sigma, St. Louis, MO, USA) antibodies. S3I-201 (100 μM; EMD Millipore, Billerica, MA) were used to inhibit Stat3 activity.

### Irradiation and flow cytometry

Cells were seeded on 6-well plates and irradiated using a 6-MV x-ray at a dose rate of 4 Gy/min (Varian Medical Systems, Palo Alto, CA, USA). For apoptosis assay using flow cytometry, cells were seeded in 6-well plates for 24 hours and transfected with different miRNA duplexes, or treated with S3I-201 or Dimethyl sulfoxide (DMSO) for 36 h. Following different treatment, cells were either were left untreated (0 Gy) or treated with 10 Gy of radiation (IR) for 48 h. After this, cells were resuspended and stained with Propidium Iodide/Annexin V/ staining kit (Thermo Fisher), and the cell populations were analyzed by a FACSCalibur Flow Cytometer (BD, San Jose, CA, USA). The percentages of dead cells are presented as the percentage of PI-positive cells.

### Statistical analysis

All statistical analysis was also carried out using SPSS 17.0 (SPSS Inc., Chicago, IL, USA) software. Data were expressed as the mean ± standard deviation of three biological replicates. Kolmogorov-Smirnov test was used to determine the normality of distribution. *t* test or Mann-Whitney U test were to compare the two groups, and *P*-values <0.05 were considered significant.

## Results

### Down-regulation of miR-124 in HER2-positive breast cancers

MiR-124 expression was significantly down-regulated in tumors compared with the matched normal tissues (Table 1, *P* = 0.002, paired *t* test). It was more likely to be down-regulated in HER-2 positive cancers than in HER-2 negative cancers (Table 1, *P* = 0.02, *t* test) and was negatively associated with tumor size (Table 1, *P* = 0.03, *t* test). In contrast, it was not significantly associated with age, disease stage, histology status, and lymph node metastasis, estrogen-receptor (ER) status, or progesterone receptor (PR) status (Table 1).

### miR-124 overexpression in HER2-positive breast cancer cells suppressed Stat3 signaling

**Figure 1:**
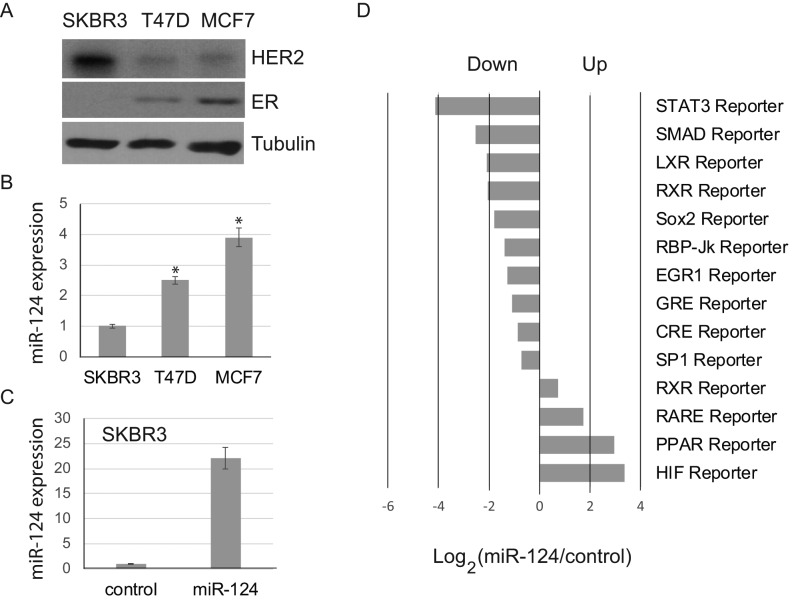
Changes in signal transduction upon microRNA-124 (miR-124) overexpression in human epidermal growth factor receptor 2 (HER-2)-positive cells. (**A**) SKBR3, T47D, and MCF7 breast cancer cells were analyzed by Western blotting for HER2 and estrogen-receptor expression. Tubulin was used as a loading control. (**B**) MiR-124 expression of in SKBR3, T47D, and MCF7 cells by realtime polymerase chain reaction. (**C**) MiR-124 expression in SKBR3 cells following transfection with miR-124 duplex or scramble control. (**D**) Significantly altered signaling pathways upon miR-124 overexpression. Histogram shows the log_2_ (fold changes) of the reporter activities of different signaling pathways. The data are presented as the mean ± standard deviation (SD) of three independent experiments. *P < 0.001.

We then investigated the HER2 and ER status in several breast cancer cell lines. Western blotting results suggested that SKBR3 cell line was HER2-positive and ER-negative (HER2+/ER-), while T47D and MCF7 cells were largely HER2-negative and ER-positive (HER2-/ER+) (Figure 1A). Interestingly, qPCR miR-124 expression was significantly lower in SKBR3 cells than in T47D and MCF7 cells (Figure 1B), suggesting a regulatory role of miR-124 inherently associated with HER2 expression.

To investigate the molecular mechanisms underlying miR-124 function in HER2-positive cells, we overexpressed miR-124 in SKBR3 cells. Transfection of miR-124 duplex in SKBR3 cells resulted in >20-fold increase in miR-124 expression compared with cells transfected with scramble control (Figure 1C). We then used a Cignal Signal Transduction Reporter Array to investigate activity changes of 50 canonical signaling pathways upon miR-124 overexpression. Based on pathway-specific transcription factor-responsive luciferase reporters, this systematic approach allowed us to identify 10 signaling pathways that were significantly suppressed and 4 signaling pathways that were significantly activated upon miR-124 overexpression (Figure 1D, >2 fold change, *P* < 0.001). MiR-124 showed the strongest inhibitory effect on the activity of Stat3 signaling pathway (Figure 1D).

### Stat3 was a direct target of miR-124 in HER2-positive breast cancer cells

**Figure 2:**
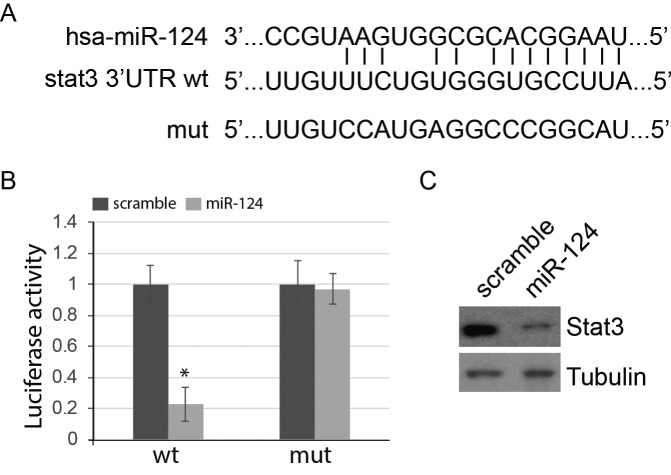
Signal transducer and activator of transcription 3 (Stat3) was a direct target of microRNA-124 (miR-124). (**A**) The potential duplexes formed between miR-124 and Stat3 3′-untranslated region region. The sequence of the mutant vector lacking the miRNA biding site is also shown. (**B**) SKBR3 were transiently transfected with the indicated constructs. Dual luciferase activity was measured 48 h post transfection. (**C**) Western blotting analysis of Stat3 expression in SKBR3 following transfection with miR-124 duplex or scramble control. The data are presented as the mean ± standard deviation (SD) of three independent experiments. *P < 0.001.

We identified Stat3 as a putative direct target of miR-124 (Figure 2A). We then performed dual-luciferase reporter assays to determine whether miR-124 could regulate Stat3 expression by directly targeting its 3′-UTR. A mutant construct with a disrupted miR-124 binding site was used as a control (Figure 2A). Compared with control, miR-124 overexpression in SKBR3 cells inhibited the transcriptional activity of luciferase reporter containing the Stat3 3′UTR, but had no effect on the activity of the mutant lacking the miR-29b binding site (Figure 2B). In line with this, miR-124 overexpression in SKBR3 cells potently reduced the expression of endogenous Stat3 protein (Figure 2C). Together, these results suggested that Stat3 was a direct target of miR-124 in HER2-positive breast cancer cells.

### Overexpression of miR-124 enhances response to irradiation in HER2-positive breast cancer

**Figure 3:**
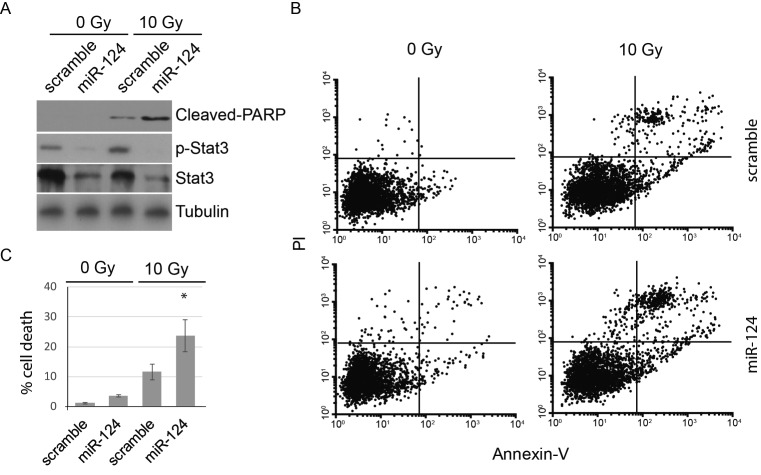
MicroRNA-124 (miR-124) overexpression enhances sensitivity to irradiation. (**A**) Western blotting for cleaved-poly (ADP-ribose) polymerase (PARP), signal transducer and activator of transcription 3 (Stat3), and p-Stat3 in SKBR3 cells transfected with miR-124 or control and treated with irradiation or not treated with irradiation. (**B**) Flow cytometry analysis of cell death in cells transfected with scramble or miR-124 and treated with irradiation. Cells were stained with Annexin V and propidium iodide. (**C**) Percentage of cell death (**B**). Data are shown as the mean ± standard deviation (SD) of three independent experiments **P < 0.001).

To test whether miR-124 overexpression enhances response to irradiation in HER2-positive breast cancer, SKBR3 cells were transfected with miR-124 or scramble duplex and then treated with 10 Gy irradiation, a dose reported previously (15). As expected, miR-124 overexpression largely reduced protein expression of Stat3 and significantly abolished Stat3 phosphorylation in SKBR3 cells (Figure 3A, *P* < 0.001). It also significantly enhanced the level of cleaved poly (ADP-ribose) polymerase (PARP) protein upon irradiation, suggesting an increased cell death index in these cells (Figure 3A, *P* < 0.001). In line with these results, flow cytometry analysis revealed that forced miR-124 expression dramatically enhanced cell death upon irradiation in SKBR3 cells (Figure 3B and C). In contrast, without irradiation miR-124 only slightly induced apoptosis.

### Stat3 and miR-124 mediates radiotherapy resistance in HER2-positive breast cancer patients

**Figure 4:**
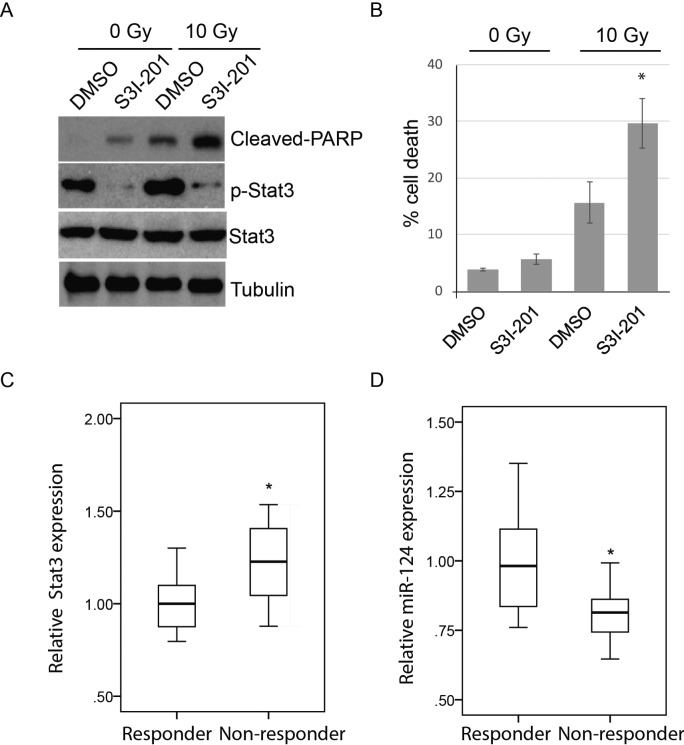
MicroRNA-124 (miR-124) expression and signal transducer and activator of transcription 3 (Stat3) is linked to radiotherapy resistance in vivo. (**A**) Western blotting for cleaved- poly (ADP-ribose) polymerase (PARP), Stat3, and p-Stat3 in SKBR3 cells treated with Dimethyl sulfoxide (DMSO) or S3I-201 and then irradiated. (**B**) Percentage of cell death determined by flow cytometry (**C**) Relative expression of Stat3 expression in recurrence-free (responder; n = 10) and relapsed (nonresponder; n = 10) human epidermal growth factor receptor 2 (HER-2)-positive breast cancer tissues determined by quantitative polymerase chain reaction. Mean expression in responders was normalized to 1. Data are presented as box-and-whisker plots. (**D**) Relative miR-124 expression in recurrence-free (responder; n = 10) and relapsed (nonresponder; n = 10) HER2-positive breast cancer tissues determined by qPCR. Interquartile ranges are presented as box-and-whisker plots. *P < 0.05.

To further validate the effects of Stat3 on radiotherapy response in HER2-positive breast cancer cells, SKBR3 cells were treated with Stat3 specific inhibitor, S3I-201, before irradiation treatment. Treatment with S3I-201 abolished Stat3 phosphorylation and significantly increased radiation-induced cell death (Figure 4A, B), supporting the fact that Stat3 is important in mediating radiotherapy resistance in HER2-positive SKBR3 breast cancer cells. To further investigate the physiological relevance of miR-124/Stat3 regulation in radiotherapy resistance, we used qPCR to measure the expression of Stat3 and miR-124 in 10 relapsed (non-responder) and 10 recurrence-free HER2-positive breast cancer patients after radiotherapy. As expected, Stat3 expression was significantly higher in non-responders than in responders (*P* = 0.032, Mann-Whitney U test), while miR-124 expression was significantly lower in non-responders than in responders (*P* = 0.002, Mann-Whitney U test).

## Discussion

In the current study, we showed that miR-124 was down-regulated in breast cancer, particularly in HER2-postive breast cancer. These results support the function of miR-124 as a tumor suppressor in breast cancer and suggest a special regulatory role of miR-124 in HER2-positive breast cancer subtypes. Forced miR-124 expression in HER-2 positive breast cancer cell line SKBR3 resulted in down-regulation of Stat3 expression, inhibition of Stat3 signaling pathway, and enhanced sensitivity to irradiation. Together, these *in vitro* and *in vivo* results suggest that miR-124/Stat3 regulation is a key factor in radiotherapy response of HER2-positive breast cancers. As Stat3 is a key upstream regulator of HER2 expression (16,17), down-regulation of miR-124 might be a driver event that causes up-regulation of Stat3 and, subsequently, cells lacking miR-124 are more likely to form HER2-positive tumors with a higher resistance to radiotherapy (18,19). This regulatory loop might explain the preferential down-regulation of miR-124 in HER2-positive breast cancer cells. We thus believe that the miR-124 could be a promising new drug target for adjuvant radiotherapy in HER2-positive breast cancers.

It is well established that Stat3 is abnormally activated in diverse tumors types (20-22). Triggered by upstream regulators with intrinsic kinase activities such as Janus kinases and epidermal growth factor receptor, Stat3 is activated by tyrosine/serine phosphorylation and relocated to the nucleus, binding to the promoters of their target genes to regulate transcription (20,21). A large variety of targets with different functions are tightly regulated by Stat3 and therefore make it a central linking point for a multitude of signaling cascades. For example, genes involved in apoptosis (*Bcl-2*, *Bcl-Xl*, and *Survivin*), cell cycle progression (*Cyclin D1*), proliferation (*c-Myc* and *Mcl-1*), and epithelial–mesenchymal transition (*TWIST*, *MMP-9*, and *MMP-7*) are all tightly regulated by Stat3 (15,16,22). Therefore, Stat3 has a crucial role in the pathogenesis and radiation resistance of breast cancer.

Consistently, previous studies have reported that miR-124 functions as an important tumor suppressor in various cancers by targeting a variety of different proteins. For example, it has been shown that miR-124 could directly target androgen receptor, enhancer of zeste homologue 2, and proto-oncogene tyrosine-protein kinase, which all contribute to prostate cancer progression and treatment resistance. MiR-124 overexpression inhibited the proliferation of prostate cancer cells in vitro and sensitized them to inhibitors of androgen receptor signaling (13). Moreover, miRNA-124 might inhibit glioma cell migration and invasion by targeting ROCK1 gene and impairing actin cytoskeleton rearrangements and reducing cell surface ruffle (14). Notably, the function of a same miRNA might vary in different cellular contexts, resulting in cancer subtype-specific regulation and response. In the current study, using a Cignal Signal Transduction Reporter Array, we systematically scanned the signaling activity changes of 50 major pathways upon miR-124 overexpression in HER2-positive cells. In addition to Stat3 signaling, we also found robust down-regulation of mothers against decapentaplegic homologue (SMAD) signaling. Transforming growth factor-β/SMAD signaling plays a critical role in the regulation of cell growth, differentiation, and development (23). Liver X receptors signaling, which is a nuclear receptor signaling pathway playing a critical role in tumor metabolism and inflammatory responses (24), was also significantly down-regulated upon miR-124 overexpression.

There are several limitations of the current study. First, the sample size was small, and further large-scale clinic studies are required to confirm our findings. Second, we only focused on HER-2 positive breast cancer, so further studies are required to validate the association between miR-124 expression, HER2 status, and radioresistance in different cancer subtypes. Interestingly, Arabkheradmand et al (11) reported an association between miR-124 down-regulation and advanced clinical stage and the presence of lymph node metastasis in Iranian patients with breast cancer, however, they found no association between microRNA expression and HER2 status. There are several explanations for the difference between our study and the study by Arabkheradmand et al: 1) The complex relation between miRNA expression and clinic clinicopathological characteristics of the patients might be different in the Iranian and Chinese Han populations. 2) The number of patients included in both studies was relatively small so the results might be affected by bias in patient selection and tumor heterogeneity. Further large-scale studies are required to validate the association between miR-124 expression, HER2 status, and radioresistance in different populations.

In conclusion, these results suggest that miR-124 is a biomarker for HER-2 positive breast cancer and a putative novel therapeutic target that can be used to overcome radiotherapy resistance.
